# A Multimodal Approach for the Risk Prediction of Intensive Care and Mortality in Patients with COVID-19

**DOI:** 10.3390/diagnostics12010056

**Published:** 2021-12-28

**Authors:** Vasileios C. Pezoulas, Konstantina D. Kourou, Costas Papaloukas, Vassiliki Triantafyllia, Vicky Lampropoulou, Eleni Siouti, Maria Papadaki, Maria Salagianni, Evangelia Koukaki, Nikoletta Rovina, Antonia Koutsoukou, Evangelos Andreakos, Dimitrios I. Fotiadis

**Affiliations:** 1 Unit of Medical Technology and Intelligent Information Systems, Department of Materials Science and Engineering, University of Ioannina, GR45110 Ioannina, Greece; bpezoulas@gmail.com (V.C.P.); Konstadina.Kourou@gmail.com (K.D.K.); papalouk@uoi.gr (C.P.); 2 Department of Biological Applications and Technology, University of Ioannina, GR45100 Ioannina, Greece; 3 Laboratory of Immunobiology, Center for Clinical, Experimental Surgery and Translational Research, Biomedical Research Foundation of the Academy of Athens, GR11527 Athens, Greece; vtriantafyllia@bioacademy.gr (V.T.); vickylampro@gmail.com (V.L.); esiouti@bioacademy.gr (E.S.); mpapadaki@bioacademy.gr (M.P.); msalagianni@bioacademy.gr (M.S.); vandreakos@bioacademy.gr (E.A.); 4 Intensive Care Unit (ICU), 1st Department of Respiratory Medicine, Medical School, National and Kapodistrian University of Athens, ‘Sotiria’ General Hospital of Chest Diseases, GR11527 Athens, Greece; e.koukaki@yahoo.gr (E.K.); nikrovina@med.uoa.gr (N.R.); koutsoukou@yahoo.gr (A.K.); 5 Department of Biomedical Research, Foundation for Research and Technology-Hellas, Institute of Molecular Biology and Biotechnology (FORTH-IMBB), GR45110 Ioannina, Greece

**Keywords:** COVID-19, artificial intelligence, dynamic modeling, risk predictors, ICU scoring index

## Abstract

Background: Although several studies have been launched towards the prediction of risk factors for mortality and admission in the intensive care unit (ICU) in COVID-19, none of them focuses on the development of explainable AI models to define an ICU scoring index using dynamically associated biological markers. Methods: We propose a multimodal approach which combines explainable AI models with dynamic modeling methods to shed light into the clinical features of COVID-19. Dynamic Bayesian networks were used to seek associations among cytokines across four time intervals after hospitalization. Explainable gradient boosting trees were trained to predict the risk for ICU admission and mortality towards the development of an ICU scoring index. Results: Our results highlight LDH, IL-6, IL-8, Cr, number of monocytes, lymphocyte count, TNF as risk predictors for ICU admission and survival along with LDH, age, CRP, Cr, WBC, lymphocyte count for mortality in the ICU, with prediction accuracy 0.79 and 0.81, respectively. These risk factors were combined with dynamically associated biological markers to develop an ICU scoring index with accuracy 0.9. Conclusions: to our knowledge, this is the first multimodal and explainable AI model which quantifies the risk of intensive care with accuracy up to 0.9 across multiple timepoints.

## 1. Introduction

The most severe pandemic of our time known as coronavirus disease 2019 (COVID-19), is consistently yielding grievous impacts in the global healthcare system. COVID-19 is caused by severe acute respiratory syndrome coronavirus type 2 (SARS-CoV-2) which was officially confirmed in January 2020 after the initial COVID-19 outbreak that took place in November 2019. Recent studies have shown that SARS-CoV-2 shares genetic similarities with its predecessor, the SARS-CoV [1], where genome sequence analysis has indicated that SARS-CoV-2 belongs to the Betacoronavirus genus, which includes the SARS-CoV, and the Middle East respiratory syndrome coronavirus (MERS-CoV) [2]. However, phylogenetic tree analysis has shown that SARS-CoV-2 is more related to Bat SARS-like coronaviruses, such as SARS-CoV and less related to MERS-CoV [3]. The uniqueness of SARS-CoV-2 lies on the fact that, unlike SARS and MERS patients, COVID-19 patients can be asymptomatic [4]. Moreover, the proliferation rate of SARS-CoV-2 is estimated to range between 2 and 2.5 times higher than SARS and MERS, a fact that strengthens its pandemic-causing potential. Furthermore, numerous variants of SARS-CoV-2 have been described so far, including more than five notable mutations [5]. COVID-19 is a highly transmittable disease, with an estimated global number of 195 million cases and 4.2 million reported deaths so far [6]. Common symptoms of COVID-19 include fever, dry cough, fatigue, and loss of taste and smell, among others [7], where the infection occurs mainly through respiratory droplets from coughing and sneezing, as well as, through contaminated surfaces [8]. Most importantly though, COVID-19 has yielded disastrous effects in the healthcare system due to the increased need for intensive care units (ICU) and ventilators.

The clinical unmet needs and open issues in COVID-19 include: (i) the development of explainable AI models for COVID-19 patients for ICU admission, and (ii) the detection of risk factors for COVID-19 onset and progression, among others. Towards this direction, several studies have been reported in the literature which utilize both statistical and artificial intelligence (AI) methods for the analysis of COVID-19 data. In [9,10], multivariate logistic regression was applied across 244 and 663 patients, respectively, to identify independent risk factors for COVID-19 mortality upon a conventional univariate analysis which highlighted disease severity, gender, white blood cell count and age as risk factors. Similar outcomes regarding the age and the C reactive protein (CRP) were reported in [11] through a multivariable Cox survival analysis. Multivariate analysis was also applied in a large retrospective study with 4404 patients [12] to identify predictors of ICU care and mechanical ventilation, as well as, in [13] to predict ICU admission across 4997 patients, highlighting procalcitonin, pulse oxygen saturation, smoking history, and lymphocyte count as significant predictors. In [14], gradient boosting trees (GBT) were trained on 1270 COVID-19 patients from Wuhan to detect prominent features for COVID-19 mortality, including disease severity, age, CRP, and lactate dehydrogenase (LDH), among others, with increased performance. In [15], the random forest (RF) algorithm was applied on clinical data from 214 patients with confirmed COVID-19 non-severe type and 148 with severe type yielding increased accuracy, as well as clinical (e.g., age, hypertension, cardiovascular disease, gender, diabetes) and laboratory (e.g., absolute neutrophil count, IL-6, and LDH) risk factors. In [16], artificial neural networks (ANNs) and bagging methods were trained across 162 hospitalized patients yielding APACHE II as a prominent risk factor with favorable sensitivity and specificity scores. In [17,18] both multivariate and machine learning algorithms, such as, the decision trees, RF, GBT and ANNs were applied to predict ICU admission and mortality across 635 patients [17] and 516 patients [18] yielding favorable predictive performance.

None of the above studies, however, have assessed the interpretability and explainability of risk predictors for ICU admission and/or mortality of hospitalized COVID-19 patients nor have explored dynamic associations among biological data across multiple time intervals through a multimodal study which utilizes risk predictors to define an ICU scoring index. In addition, the AI model validation in [13,14] was based on random splits of the data which introduce biases in performance evaluation, while the bagging methods in [14,15] introduce biases during the combination of multiple prediction outcomes which obscure the performance of the models. Finally, the application of conventional multivariate analysis (i.e., regression analysis) in [9,10,11,12,13] hamper the detection of risk predictors for ICU admission and mortality with increased statistical power due to assumptions and biases that are introduced in the analysis (e.g., the independence of the input factors).

In this work, we describe a multimodal AI approach based on an anonymized dataset of 324 hospitalized patients who have been diagnosed with COVID-19, in Greece, that includes laboratory and clinical information, as well as biological information across four time intervals. The pipeline utilizes explainable and interpretable AI models along with dynamic modeling methods to support decision making for ICU admission and/or mortality and shed light into the pathogenesis and clinical features of COVID-19. Data curation is first applied to overcome data incompatibilities and inconsistencies. Subgroup analysis is performed by dividing the curated data into four subclasses of interest based on the ICU admission and/or mortality. Gradient boosting trees (GBT) are trained on each subgroup to develop explainable AI models using concepts from coalition game theory to detect risk predictors for ICU admission and mortality, as well as, to evaluate the predictors across four time intervals. Our results highlight the importance of LDH, IL-6, IL-8, Cr, number of monocytes, lymphocyte count, and TNF as risk predictors for ICU admission and survival, as well as LDH, age, CRP, Cr, WBC, and lymphocyte count for mortality after ICU admission. These predictors were combined with those from the dynamic analysis of the biological data using dynamic Bayesian networks (DBNs) to formulate an ICU scoring index based on APACHE II [19], where the DBNs revealed notable dependencies between TNF and IL-6. To our knowledge, this is the first study that explores the interpretability of AI models and risk predictors for ICU admission and mortality of hospitalized COVID-19 patients with dynamically associated biological markers.

The paper is structured as follows. Section 2 offers a comprehensive view on the methods which were utilized in the current study, including: (i) methods for outlier detection, de-duplication, and imputation, (ii) supervised machine learning methods for AI modeling and ICU scoring, (iii) explainable methods for the detection of risk factors, and (iv) association analysis methods for the dynamic modeling of the biological data. The results of the subgroup and the overall analysis are presented in Section 3, including the explainability of the AI models, as well as the induced decision trees and the dynamic associations in four time intervals. The outcomes are discussed in Section 4 and future work in Section 5.

## 2. Materials and Methods

### 2.1. Dataset Description

Anonymized patient data were collected from 324 hospitalized patients with average age 60.65 (±14.44) who were diagnosed with COVID-19 from the 21st Department of Pulmonary Medicine, National and Kapodistrian University of Athens, in “Sotiria” Hospital for the diseases of the chest, as described in [20]. According to Appendix A Table A1, the data include demographic information, comorbidities, laboratory tests (e.g., C-reactive protein), therapies (corticosteroids and antiviral agents) as well as cytokines and interleukins measurements at four time intervals. Patient records having at least one missing value in the admission ICU date or in mortality were ignored from the analysis (110 patients). Thus, the final population included 214 patients with average age 60.93 (±15.38). Patients were categorized into four groups based on their admission in the ICU and/or mortality, where Group A included those who survived without ICU admission (131 patients, average age 55.99 (±15.1)), Group B included patients who were not admitted to the ICU but died (4 patients, average age 81 (±6.52)), Group C included those who were admitted to the ICU and survived (43 patients, average age 63.79 (±11.62)), and Group D included patients who were admitted to the ICU and died (36 patients, average age 73.81 (±9.62)).

### 2.2. Multimodal Data Analysis

**Figure 1:**
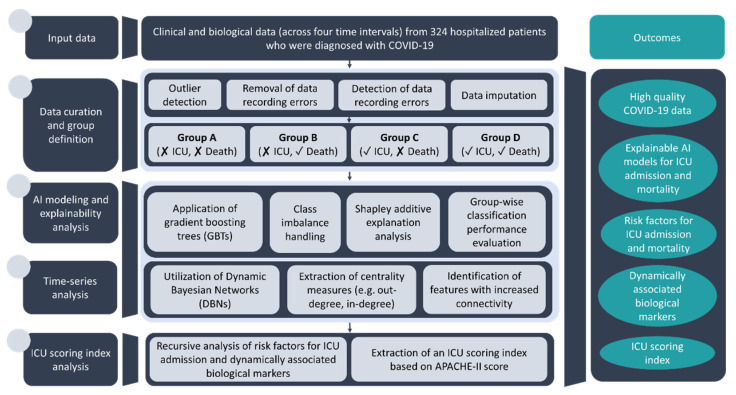
An illustration of the proposed multimodal data analysis pipeline.

The proposed pipeline is depicted in Figure 1. Data curation is first applied on the anonymized patient data to remove outliers, duplicated fields and handle missing values. The curated data are then separated into four subgroups of interest as described in Section 2.1. Gradient boosting trees (GBT) [21] are trained on the curated data from each group to develop AI models towards the classification of COVID-19 patients according to the ICU admission and mortality. Shapley additive explanation analysis [22,23] from coalition game theory is applied to identify prominent features for ICU admission and mortality. Time series analysis is utilized through the application of dynamic Bayesian networks (DBNs) to model the biological data (e.g., cytokines) in four different time intervals from all patients during their hospitalization. The most important features from the DBN analysis were combined with those from the explanation analysis to define an ICU scoring index by recursively seeking subsets of features that maximize the performance of the AI models in terms of classification accuracy. The method is applied only on the groups that involve patient admission in the ICU (i.e., Groups C and D) using the APACHE-II score as a standard score. The outcomes of the analysis include high-quality COVID-19 data, explainable AI models and risk factors for ICU admission and mortality, dynamically associated biological markers, and an ICU scoring index.

#### 2.2.1. Data Curation

A data curation pipeline presented in [24] was applied to enhance the quality of the data by removing outliers and further inconsistencies which imply recording errors. Gaussian elliptic curves [25] were used to isolate outliers by fitting multivariate gaussian distributions with different topologies (mean and standard deviation values) on the high-dimensional distributions of the input data. The features were classified according to their level of completeness into three categories, namely the “good” (no missing values and/or inconsistencies), the “fair” (≤30% missing values) and the “bad” features (>30% missing values), where the “bad” features were discarded from the analysis.

Cytokines (IL-1b, IL-6, IL-8, TNF) were grouped into four time intervals, of three days each, starting from Day 0 (i.e., hospital admission) till Day 11, where Time Interval 1 (INT1) corresponds to the average cytokine measures from Days 0 to 2, Time Interval 2 (INT2) from Days 3 to 5, Time Interval 3 (INT3) from Days 6 to 8 and Time Interval 4 (INT4) from Days 9 to 11. The kNN (k-nearest neighbors) approach [26] was used to impute the missing values only in the case of fair features to enhance the applicability and statistical power of the data. According to the kNN approach, the missing values of a given sample are imputed using the mean value from the non-missing k-nearest neighbors within the training subset, where the Euclidean distance is used to estimate the distance of a sample from its neighbors.

#### 2.2.2. Classification, AI Modeling and Explainability Analysis

##### 2.2.2.1. Problem Definition and Classification

The gradient boosting trees (GBT) classifier [21] was trained on the curated clinical data from each group (i.e., Groups A, C and D) to solve three binary classification problems, including: (i) patients in Group A, who were not admitted to the ICU and survived (outcome = 1 and 0 otherwise), (ii) patients in Group C, who were not admitted to the ICU but died (outcome = 1 and 0 otherwise), and (iii) patients in Group D, who were admitted to the ICU and died (outcome = 1, and 0 otherwise). Of note, patients in Group B were ignored from the analysis due to the small population size of the target group (4 patients). In each group, the training procedure was repeated four times using the available time-series data (IL-1b, IL-6, IL-8, TNF) in a sequential manner across the time intervals to evaluate the prediction performance of the classifier.

In this work, the GBT uses a boosting ensemble strategy which combines a set of weak regression tree learners into a much stronger one. On each boosting round, the algorithm minimizes the gradient of a logarithmic loss function to optimize the overall performance of the classifier. At step i, GBT seeks a weak tree learner, say $f_{i}\left(d\right)$, which minimizes the following cost function:

(1) $$F_{i}\left(d\right)=F_{i-1}\left(d\right)+argmin_{f}\left(\overset{n}{\underset{j=1}{\sum }}L\left({\tilde{y}}_{j},F_{i-1}\left(d_{j}\right)+f_{i}\left(d_{j}\right)\right)\right),$$

where L(y,F(d)) is the error loss function, e.g., the mean squared error (MSE), n is the number of samples and $\tilde{y}$ is the predicted value at step i.

A stratified 10-fold cross-validation procedure was used to evaluate the performance of the AI models in terms of accuracy, sensitivity, specificity, and area under the receiver operating characteristic (ROC) curve (AUC). Moreover, decision trees were trained on the whole data using an optimized version of the classification and regression trees (CART) algorithm [27] to induce interpretable rules and thus shed light into the backbone of the decision-making process using Python 3.7.6.

##### 2.2.2.2. AI Model Explainability and Interpretability

The Shapley additive explanations (SHAP) method [22,23] is used to quantify the contribution of each feature to the classification outcome in each group under investigation, where the explanation model, g, is defined as in:

(2) $$g\left(z\right)=\varphi_{o}+\overset{M}{\underset{j=1}{\sum }}\varphi_{j}z_{j},$$

where $z\in {\left\{0,1\right\}}^{N}$ is a coalition vector, M is the coalition length, and $\varphi_{j}$ is the Shapley value for the feature $x_{j}$. The latter is defined as in [22,23]:

(3) $$\varphi_{j}\left(v\right)=\overset{}{\underset{T\subseteq x\backslash \left\{x_{j}\right\}}{\sum }}\frac{\left|T\right|!\left(n-\left|T\right|-1\right)!}{n!}\left(v\left(T\cup \left\{x_{j}\right\}\right)-v\left(T\right)\right),$$

where T is a subset of a set of n-input clinical features, say $\left\{x_{1},x_{2},\ldots ,x_{n}\right\}$, v(T) is a vector of the classification outcomes given the features in T, and $v\left(T\cup \left\{x_{j}\right\}\right)$ is a vector of the classification outcomes given the features in T marginalized over the features which are not included in T.

According to (2) all possible sets of feature values need to be evaluated to calculate the exact Shapley value. An approximation to (2) was given in [28], using Monte-Carlo sampling, as in:

(4) $$\varphi_{j}\left(v\right)=\frac{1}{n}\overset{n}{\underset{i=1}{\sum }}\left(f\left(x_{j}\right)-g\left(x_{j}\right)\right),$$

where $f\left(x_{j}\right)$ is the classification outcome for x using a set of randomly selected features from z, excluding feature $x_{j}$, and $g\left(x_{j}\right)$ is the same as $f\left(x_{j}\right)$ where $x_{j}$ is not excluded. The Shapley values provide contrastive explanations of the classification outcomes which can be utilized to reveal local interpretations for the given clinical features. These explanations are based on the classification outcomes from specific training and testing instances.

In this work, the Shapley values are used instead of conventional scoring measures such as the information gain and the Gini index to preserve the properties of efficiency, symmetry, and additivity which are the fundamental properties during the evaluation of any feature importance score [23].

#### 2.2.3. Time-Series Analysis Using Dynamic Bayesian Networks (DBNs)

In this study, dynamic Bayesian networks (DBNs) were used to model the biological data (i.e., cytokines) obtained in the four time intervals from all patients during their hospitalization [29]. To perform this, conditional probability dependencies were computed between the set of cytokines, say $X=\left\{x_{1},\ldots x_{n}\right\}$, over the available time intervals. During the structure learning process, a set of conditional probability distributions (CPDs) [30] is computed, since each cytokine is a unique joint probability given the rest. The Bayesian network (BN) is defined as a pair B=(G, Θ), where the first component G is an annotated directed acyclic graph and the second one Θ represents the parameters that quantify the network. Given G and Θ, the BN, B, defines a unique joint probability distribution over X given by:

(5) $$P_{B}\left(x_{1},\ldots x_{n}\right)=\Pi_{i=1}^{n}P_{B}(x_{i}|pa\left(x_{i}\right)),$$

where $pa\left(x_{i}\right)$ denotes the parents of $x_{i}$, in G.

The DBN model is characterized by the joint distribution over all possible trajectories of a process [31] and consists of a DAG that specifies the distribution over initial states (variables at the first time slice) and a transition network for modeling the relationships of cytokines over the different time points (i.e., transition probabilities). Given a DBN model, the joint distribution over the cytokines X[0]∪X[1] is:

(6) $$P_{B}\left(x\left[0\right],\ldots ,x\left[T\right]\right)=P_{B_{0}}\left(x\left[0\right]\right)\Pi_{t=o}^{T-1}P_{B_{\to }}\left(x\left[t+1\right]|x\left[t\right]\right).$$

The key assumption when modeling a dynamic process with a DBN based on the calculation of CPDs, is that temporal dependencies among the cytokines remain stable across discrete time slices. To unwind the structure and parameters of the DBN model in this study the bnstruct R package [32] was used. The training data included the four cytokines (IL1b, IL6, IL8, TNF) that were measured at four time points. To infer the DBN model we firstly characterized the structure of the network (i.e., the graph topology for yielding the dependencies among the nodes) and subsequently we identified the parameters of each CPD (i.e., the joint distribution of each node given the values of its parents).

#### 2.2.4. ICU Scoring Index

A recursive ICU scoring index approach was developed to detect combinations of features with the highest accuracy using the APACHE-II score as a standard feature in Groups C and D. The problem is then defined as follows: For each possible set of features, say {x,y,z}, where x is a standard feature (in this case the APACHE-II), seek the features y, z, so that the input set {x,y,z} maximizes the performance of the AI models in terms of accuracy. The extracted combination is considered as an ICU scoring index which complements the APACHE-II score. The candidate features which are included in the recursive ICU approach are those with the highest importance in the DBN analysis (in terms of high degree of connectivity), as well as, in the Shapley explanation analysis (as described in Section 2.2.2.2).

## 3. Results

### 3.1. Data Curation

**Figure 2:**
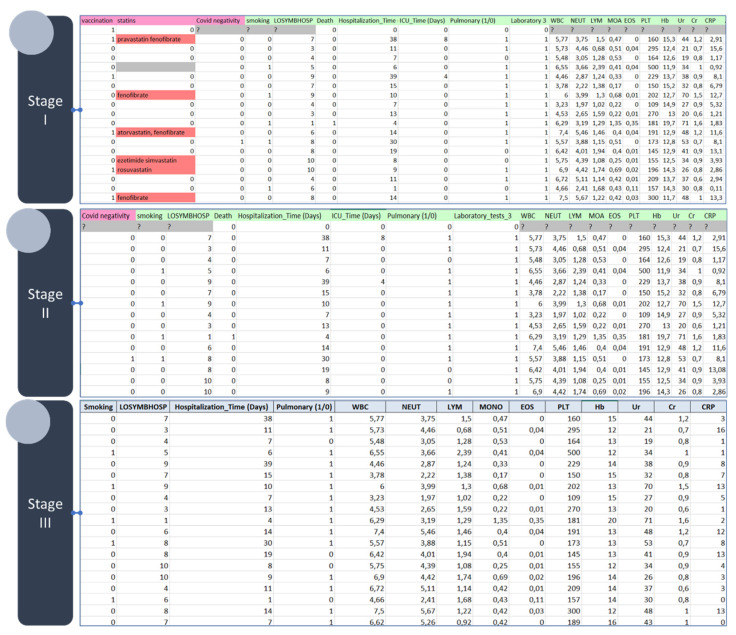
An indicative instance of the anonymized data before (on (**top**) and after (on (**bottom**)) data curation. The acronyms of the features are described in Table A1.

The initial dataset included 110 features with 324 instances. Out of 324 features, 36 were discrete, 57 were continuous and 17 features had unknown data type (i.e., mixed data types). The total number of missing values was 54.53%. After the end of the first stage of the data curation process (Stage I, Figure 2), the total number of features was 57 with 214 instances. Out of 57 features, 20 were discrete and 37 were continuous with a total of 35.38% missing values (Stage II, Figure 2). In the final stage (Stage III, Figure 2), the k-NN approach was applied for data imputation only on features with an acceptable percentage of missing values (≤40%) to increase the completeness of the data before the application of the classification models. In addition, highly associated features with the target feature, such as, the days in the ICU and the hospitalization time were removed from the analysis.

### 3.2. Subgroup Performance Evaluation of the Classification Models

**Figure 3:**
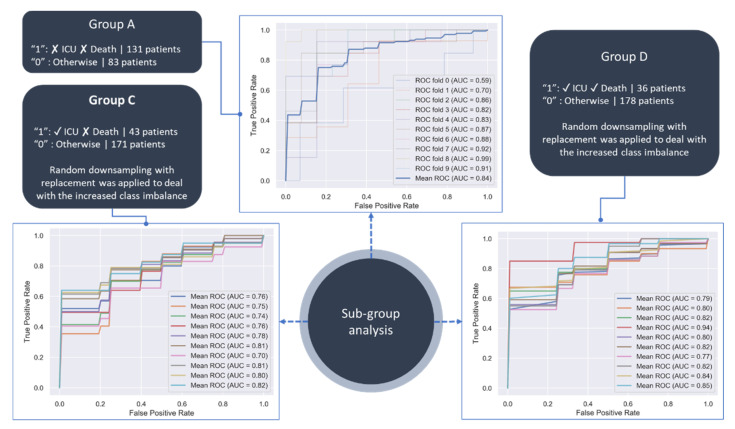
ROC curves of the GBT classifier on Groups A, C, and D in Time Interval 1.

The performance evaluation results on Groups A, C and D are summarized in Table 1 while the ROC curves for the Time Interval 1 are depicted in Figure 3. Due to the increased class imbalance in Groups C (43 targets over 171 controls) and D (36 targets over 178 controls), random downsampling with replacement was applied to yield equally numbered patients across the corresponding control and target groups [24]. More specifically, the downsampled controls were matched according to age and gender, where the downsampling ratio was set to 1:1. The overall process was repeated ten times to avoid biases during the downsampling stage. A stratified 10-fold cross validation process was applied on each round and the performance evaluation results were averaged. According to Figure 3, the classification performance was favorable in all groups (in terms of the true positive rate versus the false positive rate), where the AUC score was 0.87, 0.79, and 0.88, for Groups A, C, and D, respectively.

According to Table 1, the performance of the GBT classifier was favorable, specifically in Groups A and D. The performance of the AI model in Group A yielded an AUC 0.84 in time interval INT1, 0.84 in time intervals INT1-INT2, 0.83 in time intervals INT1-INT3, and 0.81 in time intervals INT1-INT4 towards the classification of the patients who were not admitted in the ICU and survived. The AI model in Group C was able to classify the patients who were admitted in the ICU and survived with an AUC 0.77 in INT1, 0.76 in INT1-INT2, 0.81 in INT1-INT3, and 0.8 in INT1-INT4.

Finally, the AI model in Group D classified the patients who were not admitted in the ICU and died with an AUC 0.84 in Time Interval 1, 0.84 in INT1-INT2, 0.83 in INT1-INT3, and 0.81 in INT1-INT4. It should be noted that the missing values in INT3 and INT4 affected the performance of the AI models against those trained in INT1.

### 3.3. Explainability and Interpretability of the AI Models

#### 3.3.1. Shapley Explanation Values from the Subgroup Analysis

**Figure 4:**
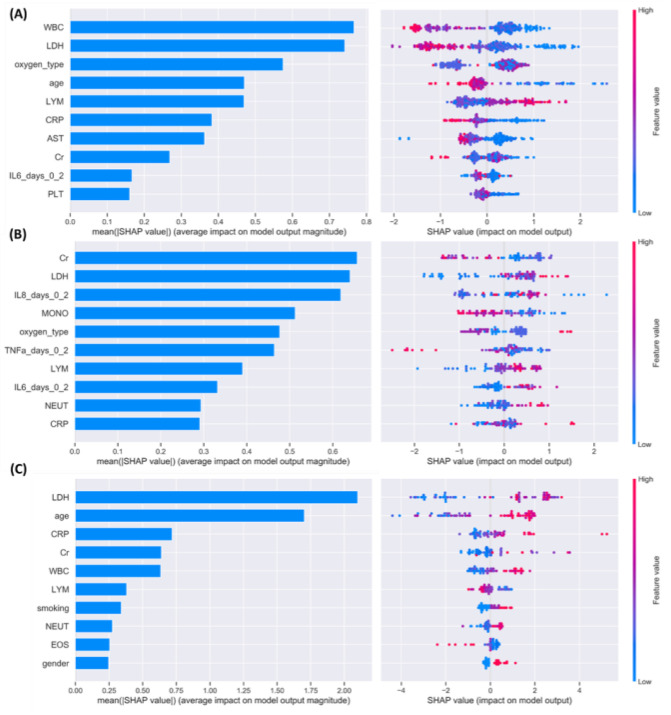
Prominent features across (**A**) Group A, (**B**) Group C, and (**C**) Group D, using the baseline data along with the cytokines from Time Interval 1 (INT1). The acronyms are described in Table A1.

The mean absolute Shapley values which quantify the average impact of each feature on the model’s output magnitude are depicted in Figure 4 (on the left subpanel) along with the Shapley values that quantify the impact of the corresponding feature on the model output (on the right subpanel). Since the objective function of the GBT classifier is set to the logistic loss, the Shapley values correspond to the log-odds. Thus, features that significantly affect the model’s output from the base value (i.e., the average model output) to higher log-odds are depicted in red whereas features that affect the average model’s output to lower log-odds are depicted in blue.

The average absolute Shapley values for Group A are depicted in Figure 4A in a descending order (on the left subpanel) along with the Shapley values (on the right) which quantify the positive or negative impact of the 10 most prominent features on the model’s output. According to Figure 4A, WBC had the highest contribution to the decision-making process by affecting the model’s output to higher log-odds for low white blood cell (WBC) values along with lactate dehydrogenase (LDH), age, C-reactive protein (CRP), aspartate aminotransferase (AST), number of platelets (PLT), and IL-6. Other features include the number of lymphocytes which affect the model’s output to higher log-odds but for higher values.

Regarding Group C, (Figure 4B) creatinine (Cr) and LDH had the highest contribution in the classification outcome, along with the IL-8, number of monocytes (MONO), oxygen type and TNF, where on one hand both low and high values of these features affect the model’s output to higher log-odds but on the other hand small LDH, IL-6, number of lymphocytes (LYM), WBC values affect the model’s output to lower log-odds. Finally, according to Figure 4C, LDH had the highest impact during decision-making in Group D, along with age, CRP, Cr, and WBC, among others, where large values for age and WBC affect the model’s output to higher log-odds but for higher values.

**Figure A1:**
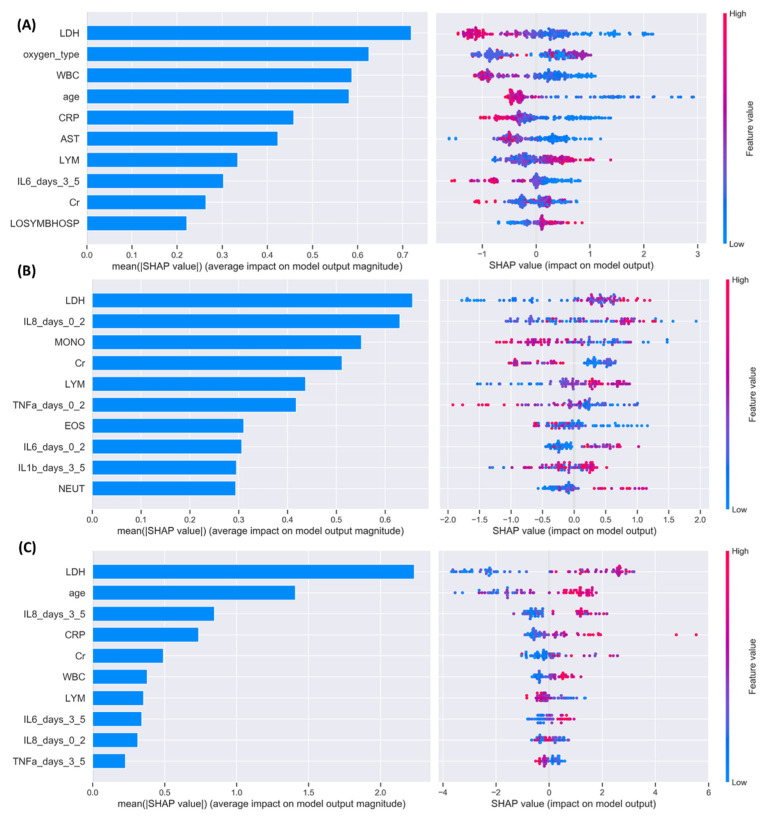
Prominent features across (**A**) Group A, (**B**) Group C, and (**C**) Group D, using the baseline data along with the cytokines from Time Intervals 1–2. The features acronyms are described in Table A1.

**Figure A2:**
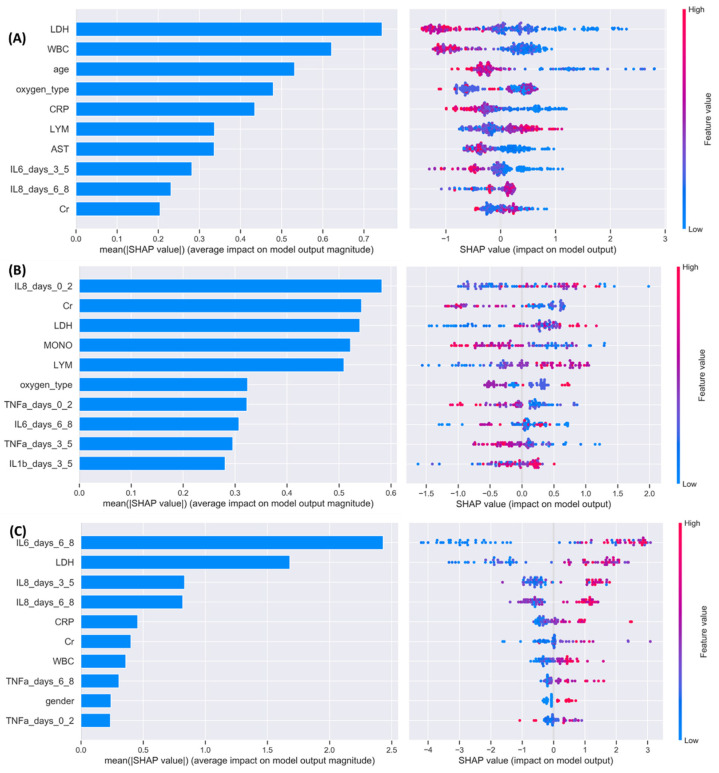
Prominent features across (**A**) Group A, (**B**) Group C, and (**C**) Group D, using the baseline data along with the cytokines from Time Intervals 1–3. The features acronyms are described in Table A1.

**Figure A3:**
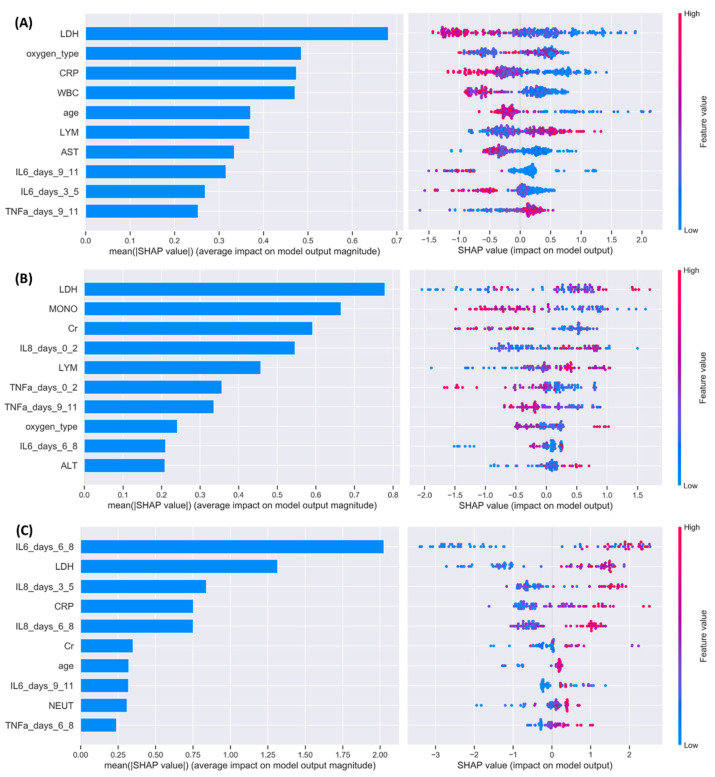
Prominent features across (**A**) Group A, (**B**) Group C, and (**C**) Group D, using the baseline data along with the cytokines from all time intervals. The features acronyms are described in Table A1.

According to Figure A1, the overall importance in Group A using the cytokines from INT2 is preserved, where the LDH, WBC, age, and CRP continue to appear as prominent, as well as IL-6 but on INT2. Regarding Group B, the features LDH, IL-8, MONO, Cr and LYM have the highest contribution to the model’s output. As far as Group D is concerned, the contribution of LDH, age, CRP, and Cr is also dominant. According to Figure A2, the overall importance in Groups A and B using the cytokines from INT1-INT3 is preserved. Regarding Group D, the contribution of LDH, IL-6 in INT3 and IL-8 in INT2 and INT3 appear to be important affecting the model’s output to higher log-odds but for higher values. Finally, according to Figure A3, the overall importance in Groups A and B using the cytokines from all time intervals is preserved with updates in the ranking order. As for Group D, the contribution of the LDH, IL-6 in INT3 and INT4 and IL-8 in INT2 and INT3 appear to be important.

**Figure 5:**
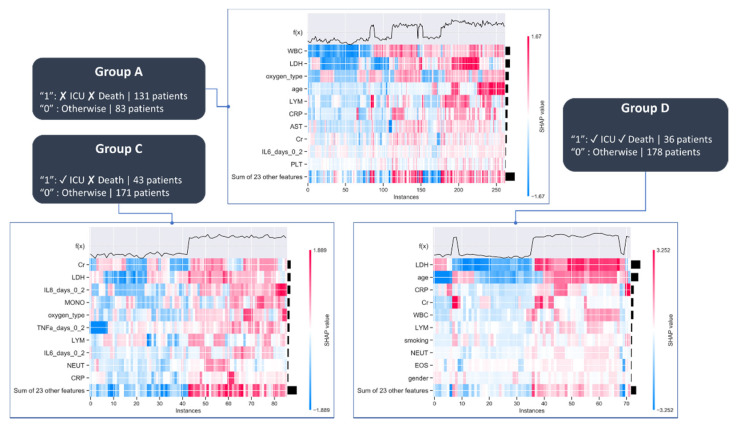
Heatmaps for Groups A, C and D, using the baseline data along with the cytokines from Time Interval 1 (INT1). The acronyms of the features are described in Table A1.

To better understand the similarities among the Shapley values of each prominent feature, heatmaps were also derived (Figure 5), where the horizontal axis depicts the instances in ascending order, the vertical axis depicts the features ranked in descending order based on their classification importance, and the color coding corresponds to the Shapley explanation value levels across the instances in the whole dataset. Hierarchical clustering was then applied based on the explanation similarity of the most prominent features to identify activation patterns among the patients.

**Figure A4:**
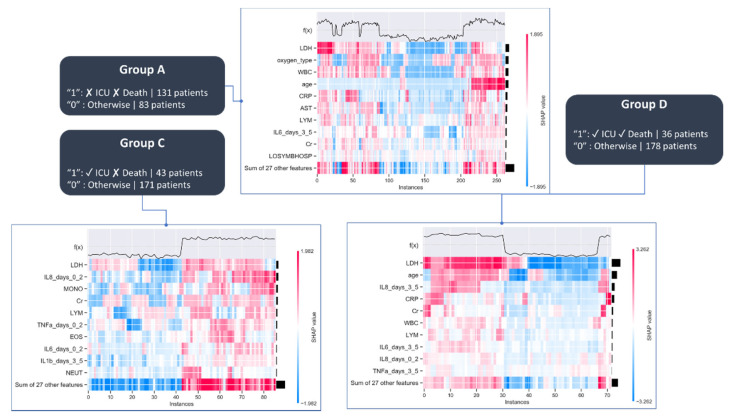
Heatmaps for Groups A, C and D, using the baseline data along with the cytokines from time intervals ΙΝΤ1–ΙΝΤ2.

**Figure A5:**
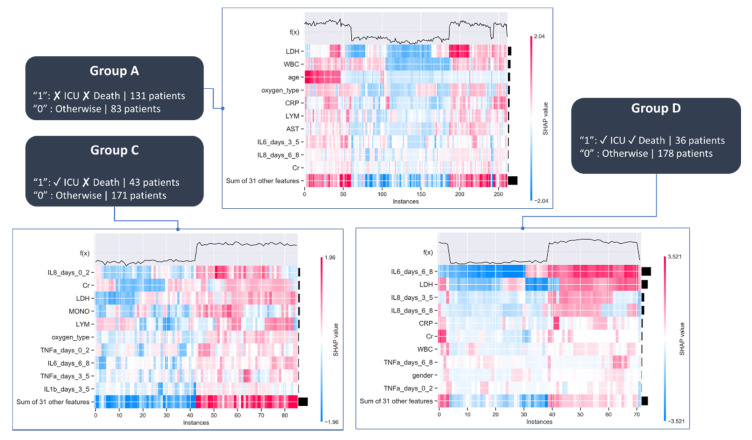
Heatmaps for Groups A, C and D, using the baseline data along with the cytokines from time intervals ΙΝΤ1–ΙΝΤ3.

**Figure A6:**
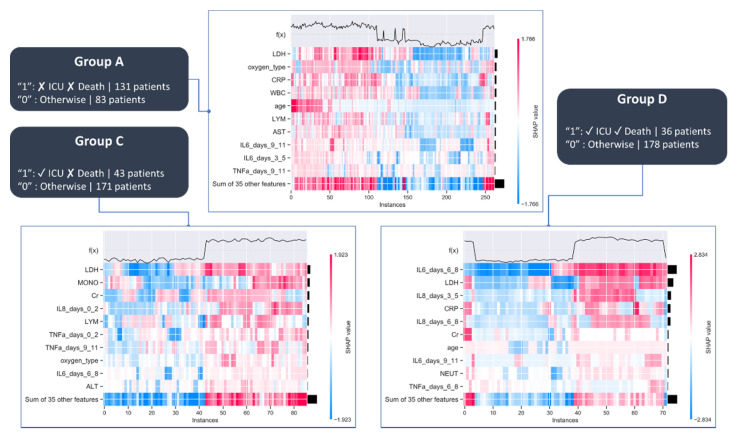
Heatmaps for Groups A, C and D, using the baseline data along with the cytokines from all time intervals.

According to Figure 5, the instances that exhibit increased explanation values using the cytokines from INT1, include the WBC, LDH, and oxygen type, which implies that these features can be used to derive homogeneous clusters and are in concordance with the feature importance plots in Figure 4. A similar pattern is observed in Group C for LDH along with IL-8, oxygen type, and TNF which are also reported in Figure 4. Regarding Group D, the LDH is an important factor for hierarchical clustering, along with the age which exhibits strong explanation similarities with the outcome. A similar behavior regarding the contribution of the prominent features from the Shapley explanation analysis to the patterns across the derived hierarchical clusters is observed in the case where the cytokine measurements from INT1-INT2 (Figure A4), INT1-INT3 (Figure A5), and INT1- INT4 (Figure A6) are used.

#### 3.3.2. Induced Decision Trees

**Figure 6:**
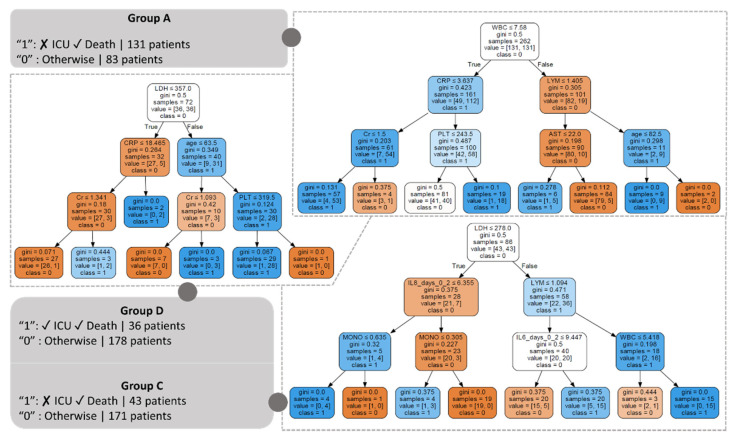
Induced decision trees across Group A, Group C, and Group D using the baseline data along with the cytokines from the Time Interval 1 (INT1; Days 0 to 2). In each case, blue color gradings denote instances which are classified as positive (i.e., outcome = “1”) whereas orange color gradings denote otherwise (i.e., outcome = “0”).

Decision trees were induced to further enhance the interpretability of the groupwise AI models by capturing the decision pathways which are involved in the decision-making process (Figure 6). Towards this direction, the CART algorithm [26] was applied on the baseline and cytokine data from each individual group and across sequential time intervals to identify critical thresholds for the prominent features, i.e., the features which are highly involved in the decision-making process, excluding Group B due to the small number of patients (Section 2.1).

According to Figure 6, the decision-making process in Group A, using the baseline data and the cytokines from INT1, is based on WBC since it is the root of the induced decision tree. The threshold 7.58 in WBC indicates a critical value that determines whether the decision will be based on CRP in case it is less than (or equal to) 7.58, where additional emphasis is given on Cr (with a critical threshold at 1.5; values less than or equal to 1.5 are classified as positive) and PLT (with a critical threshold at 243.5; instances with values larger than 243.5 are classified as positive). Otherwise, the decision-making process follows the right pathway which is based on the lymphocyte count with a critical threshold at 1.405, where in the case that this is lower than or equal to 1.405 the decision is based on AST (values less than or equal to 22 are classified as positive) or on age (values less than or equal to 82.5 are classified as positive) in the case where the lymphocyte count is higher than 1.405. It is interesting that in the case where CRP is less than (or equal to) 3.637 and Cr is less than (or equal to) 1.5, the instance is classified as positive (i.e., no admission in the ICU and survival). When CRP is larger than 3.637 and PLT is higher than 243.5, the instance is also classified as positive. In the case where WBC is higher than 7.56 the instance is classified as positive either when LYM is lower than (or equal to) 1.405 and AST is less than (or equal to) 22 or when LYM is higher than 1.404 and the age is less than (or equal to) 82.5.

As far as Group C is concerned (Figure 6), the decision-making process is based on LDH. The threshold 278 in LDH indicates a critical value that determines whether the decision will be based on IL-8 in Time Interval 1 with a threshold at 6.355, where emphasis is given on MONO (values less than or equal to 0.635 are classified as positive) in the case where IL-8 is less than or equal to 6.355 or again on MONO (values less than or equal to 0.305 are classified as positive) otherwise. Otherwise, the decision-making process follows the right pathway where emphasis is given on LYM with a critical threshold at 1.094 where in the case it is lower than 1.094 emphasis is given on IL6 at INT1 (values larger than 9.447 are classified as positive) otherwise on WBC (values less than or equal to 5.418 are classified as positive). It is interesting that in the case where LDH is less than (or equal to) 278 and IL-8 is less than (or equal to) 6.355, and MONO is less than (or equal to) 0.635 the instance is classified as positive (i.e., admission in the ICU and survival). The same occurs in the case where IL6 is larger than 6.355 and MONO is less than 0.305. However, when LDH is larger than 278 and LYM is larger than 1.094 and WBC is larger than 5.418 the instance is classified as positive. The same occurs when LYM is less than (or equal to) 1.094 and IL-6 is larger than 9.447.

**Figure 7:**
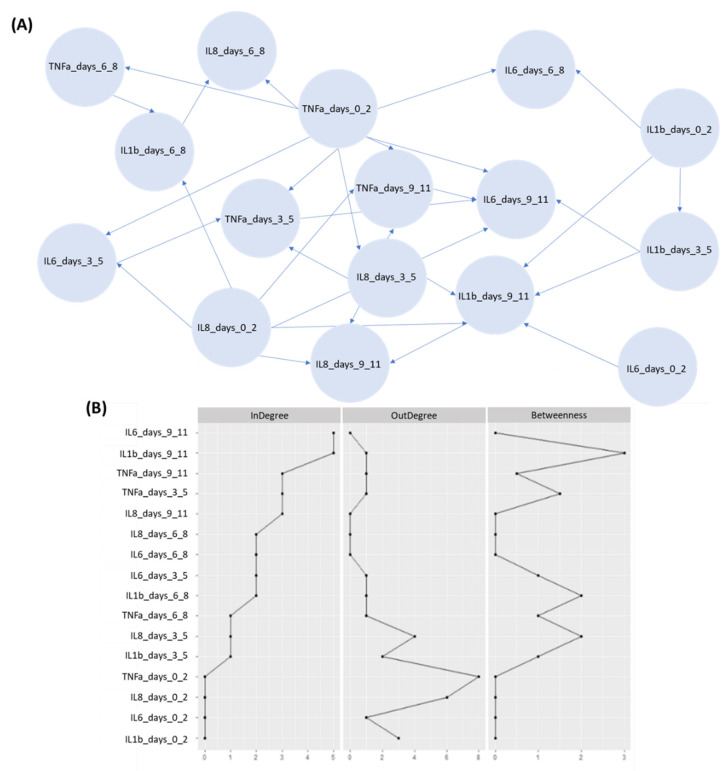
An illustration of (**A**) the derived DAG, and (**B**) the DAG-related centrality measures. The horizontal axis corresponds to the centrality indices that were used on the original scale including the zero values. The nodes have been ordered by their in-degree values.

Regarding Group D (Figure 6), the decision-making process is once more based on the LDH. The threshold 357 in LDH indicates a critical value which determines whether the decision will be based on CRP (with a critical threshold at 18.465; values larger than 18.465 are classified as positive) in the case where the LDH is less than (or equal to) 357, where emphasis is given on Cr (values larger than 1.341 are classified as positive). Otherwise, the decision-making process follows the right pathway where the decision is based on age with a critical threshold at 63.5 years, where in the case it is larger than 63.5 emphasis is given on PLT (values larger than 319.15 are classified as positive) or on Cr (values larger than 1.093 are classified as positive) otherwise. The acronyms of the features which participate in the decision-making process (Figure 7) are described in Table A1.

**Figure A7:**
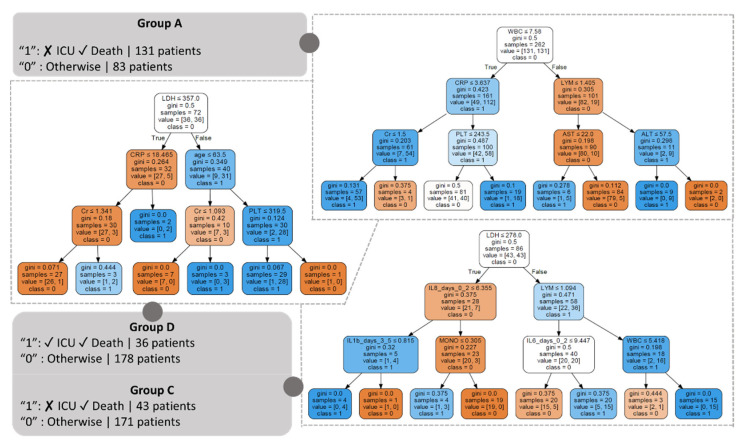
Induced decision trees across Group A, Group C, and Group D using the baseline data along with the cytokines from the time intervals ΙΝΤ1–ΙΝΤ2. Blue color gradings denote instances which are classified as positive whereas orange color gradings denote otherwise.

**Figure A8:**
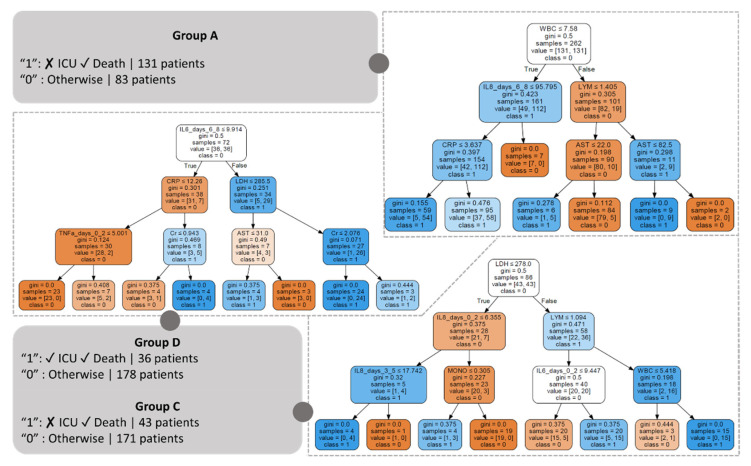
Induced decision trees across Group A, Group C, and Group D using the baseline data along with the cytokines from the time intervals ΙΝΤ1–ΙΝΤ3. Blue color gradings denote instances which are classified as positive whereas orange color gradings denote otherwise.

**Figure A9:**
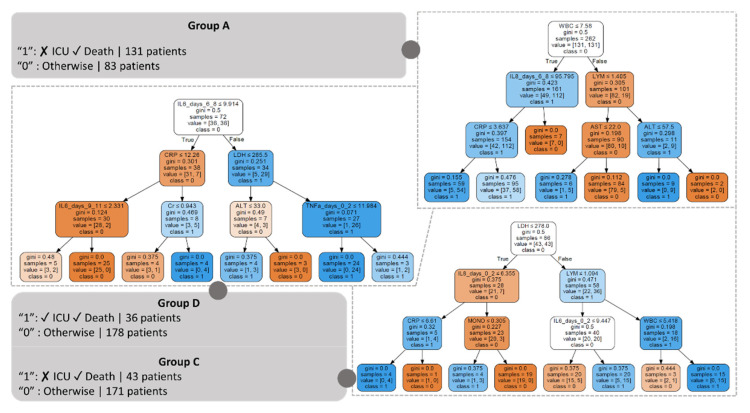
Induced decision trees across Group A, Group C, and Group D using the baseline data along with the cytokines from all time intervals. Blue color gradings denote instances which are classified as positive whereas orange color gradings denote otherwise.

The decision trees for the time intervals INT1-INT2 (Days 0 to 5), INT1-INT3 (Days 0 to 8), and INT1-INT4 (Days 0 to 11) are depicted in Figure A7, Figure A8 and Figure A9, respectively. In the case where LDH is less than (or equal to) 357 and CRP is larger than 18.465 the instance is classified as positive (i.e., admission in the ICU and death). The same occurs when CRP is larger than 18.465 and Cr is higher than 1.341. On the other hand, when LDH is larger than 357 the instance is classified as positive either when age is larger than 63.5 years and PLT is less than (or equal to) 319.5 or when age is larger than 63.5 years and Cr is larger than 1.093.

### 3.4. DBN Modeling Analysis

The DBN model obtained on the training data encodes the probabilistic relationships among the four cytokines in discrete time points as a DAG of 16 nodes and 30 edges (Figure 7). Figure 7A illustrates the inferred DBN model for the four interleukins. Figure 7B additionally presents the three measures of centrality, such as the (i) out-degree, (ii) in-degree, and (iii) betweenness within our graph [33].

The node degree corresponds to the number of connections for each node, whereas the node’s importance over information flow refer to the betweenness measure. The scaling of the graph was performed according to the raw coefficients. It should be mentioned that some of the inferred dependencies were expected when considering the ICU status of a patient who has been diagnosed with COVID-19 and has or has not died subsequently.

IL-6 protein exhibits high degree of connectivity especially within the last time points in the DBN model while the betweenness centrality is also high for this variable in the last time point. We can also observe that potential connectivity appears between the interleukins IL-6 and IL-8 over time, as well as, between TNF cytokine and IL-6 interleukin.

### 3.5. ICU Scoring Index Analysis

The recursive method described in Section 2.2.4 yielded the set of features {APACHE II, IL1b_days_0_2, IL8_days_0_2} as the one with the best performance in Group C (0.77 accuracy) using only the cytokines from INT1 (the accuracy was 0.76 using only APACHE-II score). According to Table 2, the set of features {APACHE II, IL6_days_0_2, IL1b_days_3_5}, {APACHE II, IL6_days_3_5, TNF_days_3_5}, and {APACHE II, TNF_days_3_5, TNF_days_6_8} achieved the best performance with accuracy scores 0.78, 0.79 and 0.81 in INT1-INT2, INT1-INT3, and INT1-INT4, respectively. As far as Group D is concerned, the set of features {APACHE II, IL1b_days_0_2, IL6_days_0_2} achieved the best performance with accuracy 0.8 (the accuracy was 0.77 using only the APACHE-II score). Regarding the rest of the time intervals, the set of features {APACHE II, IL6_days_0_2, IL6_days_3_5}, {APACHE II, IL6_days_0_2, TNF_days_0_2}, and {APACHE II, IL8_days_3_5, IL1b_days_6_8} achieved the best performance with accuracy scores 0.855, 0.869 and 0.902, in INT1-INT2, INT1-INT3, and INT1-INT4, respectively. This implies that APACHE-II can be combined with IL-8, as well as, with IL-6, TNF and IL-1b to yield a scoring index which could serve as a primary indicator of the severity of the disease during the admission of patients with SARS-CoV-2 in the ICU.

## 4. Discussion

In this work, we developed a multimodal data analytics pipeline which utilizes explainable and interpretable AI models along with dynamic modeling methods on curated clinical data to understand the pathogenesis and risk factors of COVID-19 regarding ICU admission and mortality. The extracted risk factors for ICU admission and/or mortality were combined with the APACHE-II score, which has been reported in [34,35] as one of the most contributory variables for the risk prediction of COVID-19, to develop an ICU scoring index with accuracy 0.9 based on IL-6, IL-8, IL-1b and TNF and thus quantify the severity of the disease. Our results highlight the importance of LDH, age, CRP, WBC, IL-6, IL-8, Cr, number of monocytes, lymphocyte count, and TNF as risk predictors for ICU admission (and survival) and mortality in the ICU, among others (the acronyms are described in detail in Table A1). A similar picture is observed in the case of time intervals INT1-INT2, INT1-INT3, and INT1-INT4 with updates in the ranking order.

The proposed method focuses on the detection of explainable risk predictors for ICU admission and/or mortality based on high quality clinical and biological data (Figure 2), as well as, to the identification of an ICU scoring index which complements the APACHE-II score compared to the workflows that are presented in [17,18] and focus only on the extraction of risk factors. Furthermore, the proposed approach avoids the application of conventional multivariate regression analysis such as in [9,10,11,12,13] since these types of methods are based on statistical assumptions regarding the independence of the input factors and thus reduce the statistical power of the outcomes. In addition, the AI model validation process is not based on random splits of the data as in [13,14] nor on the application of bagging methods as in [14,15,17] which introduce biases during the assembly stage and the performance evaluation of the AI models. The identified risk factors for ICU admission and mortality in the ICU are in line with those presented in the literature (Table 3), including the LDH, CRP, IL-6, IL-8, lymphocyte count, and WBC, among others. To further highlight the prediction performance of the proposed AI model we compared it against four other machine learning schemas, including the logistic regression (LR), the support vector machines (SVM), the AdaBoost and the naïve Bayes (NB). The prediction performance results are summarized in Table A2. According to Table A2, the GBT had the best performance in all cases and for all groups under investigation.

The classification performance of the AI model in Group A regarding the patients who were not admitted in the ICU and survived was favorable (Table 1, Figure 3), where the classification performance in time intervals INT3 and INT4 was less than INT1 since patients in Group A did not remain in the hospital for many days and thus most of the cytokine measures in the future time intervals were missing. Regarding Group C, the performance of the AI model in INT2 was lower than INT1 due to the higher percentage of missing cytokines in INT2 (Table 1, Figure 3). The same occurred in INT4 when compared against INT3. The AI model in Group D was not affected by the missing cytokine measurements, as in the previous groups, since the number of patients who were submitted in the ICU and died was small and easily separable thus the impact of the missing cytokines in future time intervals was irrelevant in this case.

Regarding the findings of the explainability analysis from Section 3.3.1 (Figure 4 and Figure 5), the importance of LDH has been confirmed in [35,36,37,38,39,40] as an independent risk factor for the severity and mortality of COVID-19. In addition, IL-6 has been linked to severity and duration of hospitalization in [20]. Furthermore, IL-6 has been identified in [41] as a disease severity predictor for COVID-19 and in [42] as a key factor, among numerous cytokines and chemokines, the treatment of which can reduce mortality in COVID-19 patients. The importance of IL-8 has been highlighted in [43] along with other circulating cytokines, including IP-10 (CXCL10), MCP1 (CCL2), and RANTES (CCL5). CRP levels have been positively associated with the severity of COVID-19 in [44,45,46,47,48], where the elevated levels of CRP and IL-6 have been proposed as predictors for mechanical ventilation in COVID-19 [47]. Age is a major predictor of mortality especially in older patients and has been considered as a key factor for the definition of various scoring systems for COVID-19 [48,49]. The importance of IL-6 and IL-8 has been also stated in [50] in which the profiling of serum cytokines IL-6 and IL-8 have been identified as disease severity predictors for COVID-19. Increased cytokine levels, including TNF and IL-6 have been also reported in [20,51] as risk factors for severity and mortality in COVID-19. Creatinine has been identified as an independent risk factor for predicting adverse outcomes in COVID-19 patients [52] but has been reported only on a few case studies in the literature. The diagnostic and predictive role of the lymphocyte-to-monocyte ratio, the neutrophil-to-lymphocyte ratio, and the platelet-to-lymphocyte ratio in COVID-19 patients has been reported in [53].

The induced decision trees (Figure 6) confirm the importance of LDH and WBC counts in the decision-making process across Groups A, C, and D. Furthermore, the decision trees have highlighted the importance of CRP [54] and number of lymphocytes [53] as prominent factors for mortality in COVID-19. The clinical significance of the WBC morphology has been noted in [55] and its diagnostic and prognostic value in COVID-19 patients has been highlighted in [56]. The profiling of cytokines has also revealed IL-8 (apart from IL-6) as a disease severity predictor which is in line with the findings reported in [41,48,57]. Regarding the number of neutrophils (NEUT), the neutrophil-to-lymphocyte ratio has been found as an independent risk factor for mortality in hospitalized patients with COVID-19 [58]. The AST and ALT levels have been also associated with the mortality in COVID-19 patients [59]. In addition, the PLT count is related with the prediction of severe illness in COVID-19 [60].

Critical thresholds of the above risk predictors were identified by the induced decision trees using the baseline data and the cytokines from INT1 (Figure 6). More specifically, in Group A, the threshold 7.58 in WBC counts determines whether the decision will be based on CRP in case it is less than (or equal to) 7.58, where emphasis is given to Cr and PLT or LYM count, AST and age. Regarding Group C, the threshold 278 in the LDH determines whether the decision will be based on IL-6, and the number of monocytes in case it is less than (or equal to) 278 or on LYM, IL-6 and WBC, otherwise. As for Group D, the threshold 357 in LDH indicates a critical value which determines whether the decision will be based on CRP and Cr in case it is lower than (or equal to) 257, or on age, Cr and PLT count otherwise.

To capture an overall picture of the prominent risk factors for ICU admission and mortality a multiclass problem was also investigated using a random forests classifier which was trained on the 214 patients to solve a four-class classification problem, where Class “0” denotes the patients who belong in Group A, Class “1” those in Group B, Class “2” those in Group C, and Class “3” those in Group D. Feature ranking was measured based on the Gini index [26] across the total number of instances. The obtained classification performance was 0.71. The set of prominent features include the CRP, WBC, LDH, IL-6 in INT2 and INT3, AST and age, which are in line with the findings in the subgroup analysis, along with NEUT (number of neutrophils) and ALT (alanine transaminase) which appear to be important, as well.

According to our results, IL-6 which has been characterized as a prognostic marker, exhibits high degree of connectivity (in-degree) within our network (Figure 7) which implies its increased association, in terms of causality, with other biological markers. Hence, in the clinical practice we can detect all the causal relationships among important nodes for the progression of the disease. The study of IL-6 in patients with severe COVID-19 pneumonia revealed its high association with outcomes, such as, death and respiratory failure [61]. These findings are corroborated with the connections depicted in the DBN model and the centrality measures listed in Figure 7 for this certain predictor. Moreover, functional associations of this protein should be further investigated towards the improvement of the risk prediction of severe COVID-19 comorbidities and death. We can also observe that the proposed model confirms the relationships among IL-6 and IL-1b since they exhibit a high degree of connectivity and betweenness centrality.

The DBN model yielded notable dependencies between TNF and IL-6 (Figure 7), which support its robustness and generalizability since the analysis has identified both as predictors for monitoring COVID-19 patients. Besides its generalizability, the DBN model identified interesting dependencies between TNF and IL-8 which have not yet been deeply investigated in the literature. Furthermore, the TNF cytokine presents a high in-degree in the proposed DAG, revealing its potential and connection with other cytokines during the disease progression. During the pandemic, anti-TNF therapy has been studied for its potential as a treatment for COVID-19 besides the anti-IL-6 receptor therapy [62]. According to the results of the COVID-19 Global Rheumatology Alliance registry, anti-TNF therapy was associated with a lower rate of hospital admission and death. To this end, the elevation of concentrations in both TNF and IL-6 should be monitored for avoiding further inflammation in COVID-19 patients and thereby avoid the need for ICU. In the case of patients with autoimmune diseases, treatments are based on drugs such as anti-TNF agents and interleukin-6 receptor blockers, thus favoring the onset of infections by blocking the signal transduction from the cell surface receptors to the nucleus [63].

The ICU scoring index analysis which was conducted in Groups C and D using only the cytokines that were identified as important from the DBN analysis and the explainability analysis, yielded significant risk predictors for ICU admission and mortality. More specifically, the proposed method was able to recursively identify the APACHE-II, IL-1b, and IL-8 as those contributing most to the classification accuracy in Group C and for INT1, which suggests that these features can be used as disease severity predictors for ICU scoring and thus can determine the admission of hospitalized patients with COVID-19 in the ICU. In addition, the APACHE-II, IL-1b, and IL-6 were also highlighted as risk predictors for ICU admission and mortality in Group D. Regarding the rest of the time interval combinations in Group C, it is interesting to note that cytokines measured in previous time intervals continue to remain prominent in future time intervals as well (e.g., IL-6 from INT1 remains important in INT1-INT2, IL-6 from INT2 preserves its importance in INT1-INT3, and TNF from INT2 in INT1-INT4). As for Group D, a similar pattern is observed, where IL-6 from INT1 remains important even when the cytokines from INT1-INT2 and INT1-INT3 are used, TNF from INT1 remains important when using INT1-INT3, and IL-8 from INT2 remains prominent in the analysis even when using INT1-INT4 along with IL-1b measured in INT3.

Altogether, our results from the baseline data and cytokines from INT1 highlight the importance of LDH, IL-6, IL-8, Cr, number of monocytes, lymphocyte count, and TNF as risk predictors for ICU admission and survival, as well as LDH, age, CRP, Cr, WBC, and lymphocyte count as risk predictors for mortality after ICU admission, among others. Based on DBN modeling the prediction of probable and reasonable trajectories was provided over time, considering the measurement of the four cytokines in discrete time points. Moreover, the model revealed the probabilistic relationships among risk factors of COVID-19 regarding ICU admission and mortality. For instance, we found that IL-6 influences the levels of TNF in the last time point and more dependencies were evidenced over time between TNF and IL-8. The most important features from the DBN analysis were finally combined with the risk predictors from Shapley explanation analysis to extend the clinical impact of the APACHE-II score towards the development of a scoring index based on IL-6, IL-8, IL-1b and TNF during the admission of hospitalized COVID-19 patients in the ICU across different time intervals of the disease.

## 5. Conclusions

This work focused on the development of a multimodal and explainable AI model to predict the risk of intensive care and mortality across multiple timepoints with accuracy 0.79 and 0.81, respectively. Modeling COVID-19 progression through DBNs by cytokines’ measurements over time identified notable dependencies among clinical and biological markers, where most of them are biomarkers of inflammation, including the IL-8, IL-6, CRP, LDH, and TNF. This implies that patients with critical inflammation levels have a higher risk for ICU admission and mortality. These biomarkers were combined with the APACHE-II score to formulate a highly robust ICU scoring index with accuracy up to 0.9.

The limitations of the current study include the small size of the available data and the increased percentage of missing values from the available cytokine data across the four time intervals (Days 0 to 2, 3 to 5, 6 to 8, 9 to 11), where Day 0 corresponds to the admission day in the hospital. Towards this direction, we plan to utilize the proposed multimodal data analysis pipeline across a larger sample hospitalized COVID-19 patients in the future and analyze follow-up data across more time points, further enhancing the statistical power of the outcomes. In addition, we plan to include clinical information in the timing where the patient started to show symptoms of COVID-19 to the time he/she presents to the hospital. We also plan to explore the fusion of the available clinical and laboratory related data with RNA-sequencing (transcriptomic) data and/or imaging-based features to shed light into the genetic mechanisms and underlying associations of the existing risk prediction factors of COVID-19 for ICU admission and mortality. We plan finally to include in our workflow additional measurements for the ratio of arterial oxygen partial pressure (PaO_2_) to fractional inspired oxygen (FiO_2_) across multiple time intervals and investigate its prediction value towards the selection of targeted therapeutic treatments for COVID-19.

## Figures and Tables

**Table 1:** Performance evaluation results across sequential time intervals for Groups A, C, and D. Group B was ignored due to the small number of patients (INT1: Days 0 to 2, INT2: Days 3 to 5, INT3: 6 to 8 and INT4: Days 9 to 11).

**INT1**				
**Groups**	**Accuracy**	**Sensitivity**	**Specificity**	**AUC**
Group A *	0.77	0.77	0.71	0.84
Group C **	0.73	0.73	0.75	0.77
Group D **	0.77	0.77	0.78	0.83
**INT1-INT2**				
**Groups**	**Accuracy**	**Sensitivity**	**Specificity**	**AUC**
Group A *	0.79	0.79	0.71	0.84
Group C **	0.72	0.72	0.72	0.76
Group D **	0.77	0.77	0.77	0.84
**INT1-INT3**				
**Groups**	**Accuracy**	**Sensitivity**	**Specificity**	**AUC**
Group A *	0.77	0.77	0.70	0.83
Group C **	0.78	0.77	0.80	0.81
Group D **	0.79	0.78	0.81	0.86
**INT1-INT4**				
**Groups**	**Accuracy**	**Sensitivity**	**Specificity**	**AUC**
Group A *	0.77	0.77	0.69	0.82
Group C **	0.77	0.77	0.77	0.80
Group D **	0.81	0.80	0.82	0.85

* A stratified 10-fold cross validation procedure was used. ** Random downsampling with replacement was applied to match the control group with the target group due to the increased class imbalance (Section 2.1).

**Table 2:** Set of features with the highest accuracy for ICU scoring.

**Group C**		
**Time intervals**	**Set of features**	**Accuracy**
**-**	{APACHE II}	0.76
**INT1**	{APACHE II, IL1b_days_0_2, IL8_days_0_2}	0.77
**INT1-INT2**	{APACHE II, IL6_days_0_2, IL1b_days_3_5}	0.78
**INT1-INT3**	{APACHE II, IL6_days_3_5, TNF_days_3_5}	0.79
**INT1-INT4**	{APACHE II, TNF_days_3_5, TNF_days_6_8}	0.81
**Group D**		
**Time intervals**	**Set of features**	**Accuracy**
**-**	{APACHE II}	0.77
**INT1**	{APACHE II, IL1b_days_0_2, IL6_days_0_2}	0.80
**INT1-INT2**	{APACHE II, IL6_days_0_2, IL6_days_3_5}	0.85
**INT1-INT3**	{APACHE II, IL6_days_0_2, TNF_days_0_2}	0.87
**INT1-INT4**	{APACHE II, IL8_days_3_5, IL1b_days_6_8}	0.90

**Table 3:** Comparison with existing state-of-the-art studies.

Study	Dataset	Method	Outcomes
[9]	Electronic medical records with symptoms, signs, and laboratory findings from 244 hospitalized COVID-19 patients in China.	Multivariate logistic regression analysis was used to identify risk factors for mortality.	**Risk factors for mortality:** disease severity, gender, white blood cell count and age as risk factors, C reactive protein (CRP).
[10]	Clinical and laboratory data from 663 COVID-19 patients in China.	Multivariate logistic regression analysis to model the disease severity.	**Risk factors for disease severity:** sex, disease severity, expectoration, muscle ache, and decreased albumin.
[11]	Clinical data from 3894 COVID-19 patients in Italy.	Machine learning (random forest) and Cox survival analysis were used to identify risk factors for mortality in the hospital.	**Risk factors for mortality:** impaired renal function, elevated C-reactive protein, and advanced age.
[12]	Medical records from 4404 COVID-19 patients in China.	Exploratory multivariate analysis was applied to identify predictors of ICU care and mechanical ventilation.	**Risk factors for ICU admission and death:** lower oxygen saturations, high respiratory rates.
[13]	Electronic medical records from 4997 COVID-19 patients in the U.S.	Multivariate logistic regression was applied to predict ICU admission and death.	**Risk factors for ICU admission:** lactate dehydrogenase, procalcitonin, pulse oxygen saturation, smoking history, lymphocyte count. **Risk factors for mortality:** heart failure, procalcitonin, lactate dehydrogenase, chronic obstructive pulmonary disease, pulse oxygen saturation, heart rate, age.
[14]	Clinical and laboratory data from 1270 COVID-19 patients in the U.S.	Multi-tree extreme gradient boosting (XGBoost) was used to detect prominent features for COVID-19 mortality.	**Risk factors for mortality:** disease severity, age, levels of high-sensitivity C-reactive protein (hs-CRP), lactate dehydrogenase (LDH), ferritin, and interleukin-10 (IL-10).
[15]	Clinical and laboratory data from 214 COVID-19 patients in China.	Random forest (RF) algorithm to differentiate severe and no severe COVID-19 clinical types based on multiple medical features and provide reliable predictions of the clinical type of the disease.	**Risk factors for disease severity:** age, hypertension, cardiovascular disease, gender, diabetes, absolute neutrophil count, IL-6, LDH.
[16]	Clinical and laboratory data from 162 hospitalized COVID-19 patients in Israel.	Artificial neural networks (ANNs) and bagging methods to predict the risk for critical COVID-19.	**Risk factors:** white blood cell count, time from symptoms to admission, oxygen saturation and blood lymphocytes count, APACHE II.
[17]	Clinical and laboratory data from 635 COVID-19 patients in the U.S.	Multivariate and machine learning algorithms, such as, the decision trees, RF, GBT and ANNs were applied to predict risk factors for ICU admission and mortality.	**Risk factors for mortality:** age, procalcitonin, C-creative protein, lactate dehydrogenase, D-dimer, and lymphocytes. **Risk factors for ICU admission:** procalcitonin, lactate dehydrogenase, C-creative protein, pulse oxygen saturation, temperature, and ferritin.
[18]	Clinical and laboratory data from 516 COVID-19 patients in China.	To produce models of mortality or criticality (mortality or ICU admission) in a development cohort using machine learning algorithms (e.g., XGBoost, Random Forests).	**Risk factors for mortality:** age, diastolic pressure, O2 Sat, BMI, AST, creatinine, CRP, ferritin, platelet, RDW, WBC. **Risk factors for criticality:** age, O2 Sat, ALT, AST, creatinine, CRP, ferritin, platelet, RDW, WBC, neutrophil/lymphocyte ratio.
Proposed	Clinical and biological data across four time points from 324 COVID-19 patients in Greece.	A multimodal data analytics pipeline which utilizes explainable and interpretable AI models along with dynamic modeling methods to identify risk factors of COVID-19 regarding ICU admission and mortality and develop an ICU scoring index.	**Risk factors for mortality:** LDH, IL-6, IL-8, Cr, number of monocytes, lymphocyte count, and TNF. **Risk factors for ICU admission and survival:** LDH, age, CRP, Cr, WBC, lymphocyte count for mortality in the ICU. These risk factors were combined with dynamically associated biological markers to develop an ICU scoring index with accuracy 0.9.

## Appendix A

**Table A1:** A summary of the features that participated in the analysis (after data curation).

Feature	Description	Value Range
Age	-	[18, 91]
Gender	-	[0, 2]
Comorbidities (presence)	-	[0, 1]
Diabetes Type I	-	[0, 1]
Diabetes Type II	-	[0, 1]
Dyslipidemia	-	[0, 1]
Hypertension	-	[0, 1]
Thyroidism	-	[0, 1]
COPD	Chronic obstructive pulmonary disease	[0, 1]
Atrial fibrillation	-	[0, 1]
Allergic rhinitis	-	[0, 1]
Asthma	-	[0, 1]
Others	Presence of any other comorbidities	[0, 1]
APACHE II	-	[0, 20]
Vaccination	-	[0, 1]
Smoking	-	[0, 2]
LOSYMBHOSP	Day interval from the first symptom until the admission to the hospital	[1, 29]
WBC	White blood cell count	[2.53, 20]
NEUT	Neutrophils	[0.97, 17.32]
LYM	Lymphocytes	[0.14, 3.09]
MONO	Monocytes	[0.04, 1.35]
EOS	Eosinophils	[0, 0.71]
PLT	Platelets	[52, 560]
Hb	Hemoglobin	[8, 73]
Cr	Creatinine	[0.5, 3.2]
CRP	C-reactive protein	[0.11, 29]
AST	Aspartate Aminotransferase	[15, 380]
ALT	Alanine Aminotransferase	[8, 223]
LDH	Lactate Dehydrogenase	[38, 1394]
Oxygen type	0: no oxygen, 1: ventilator, 2: oxygen (mask, nasal canula), 3: none of the above, 4: non-invasive (CPAP, BIPAP), 5: high flow	[0, 5]
IL1b_days_0_2	Interleukin 1 beta in Time Interval 1—INT1 (averaged across Days 0–2)	[0.028, 1.82]
IL6_days_0_2	Interleukin 6 in Time Interval 1—INT1 (averaged across Days 0–2)	[0.137, 60.891]
IL8_days_0_2	Interleukin 8 in Time Interval 1—INT1 (averaged across Days 0–2)	[0.287, 90.426]
TNF_days_0_2 (or TNFa_days_0_2)	Tumor necrosis factor (alpha) in Time Interval 1—INT1 (averaged across Days 0–2)	[0.963, 17.23]
IL1b_days_3_5	Interleukin 1 beta in Time Interval 2—INT2 (averaged across Days 3–5)	[0.107, 1.58]
IL6_days_3_5	Interleukin 6 in Time Interval 2—INT2 (averaged across Days 3–5)	[0.06, 58.841]
IL8_days_3_5	Interleukin 8 in Time Interval—INT2 (averaged across Days 3–5)	[1.635, 111.816]
TNF_days_3_5 (or TNFa_days_3_5)	Tumor necrosis factor (alpha) in Time Interval 2—INT2 (averaged across Days 3–5)	[1.191, 21.086]
IL1b_days_6_8	Interleukin 1 beta in Time Interval 3—INT3 (averaged across Days 6–8)	[0.246, 0.968]
IL6_days_6_8	Interleukin 6 in Time Interval 3—INT3 (averaged across Days 6–8)	[0.248, 17.788]
IL8_days_6_8	Interleukin 8 in Time Interval 3—INT3 (averaged across Days 6–8)	[2.519, 115.776]
TNF_days_6_8 (or TNFa_days_6_8)	Tumor necrosis factor (alpha) in Time Interval 3—INT3 (averaged across Days 6–8)	[1.297, 10.018]
IL1b_days_9_11	Interleukin 1 beta in Time Interval 4—INT4 (averaged across Days 9–11)	[0.055, 2.291]
IL6_days_9_11	Interleukin 6 in Time Interval 4—INT4 (averaged across Days 9–11)	[0.0002, 176.207]
IL8_days_9_11	Interleukin 8 in Time Interval 4—INT4 (averaged across Days 9–11)	[0.652, 56.1]
TNF_days_9_11 (or TNFa_days_9_11)	Tumor necrosis factor (alpha) in Time Interval 4—INT4 (averaged across Days 9–11)	[1.202, 9.408]
Group *	0: Group A, 1: Group B, 2: Group C, 3: Group D	[0, 3]

* Group A: patients who were not admitted to the ICU and survived, Group B: patients who were not admitted to the ICU but died, Group C: patients who were admitted to the ICU but survived, Group D: patients who were admitted to the ICU and died.

**Table A2:** Classification accuracies of different types of classifiers (LR: logistic regression, SVM: support vector machines, NB: naïve Bayes, AdaBoost: adaptive boosting, GBT: gradient boosting trees) for Groups A, C, and D across sequential time intervals. Group B was ignored due to the small number of patients (INT1: Days 0 to 2, INT2: Days 3 to 5, INT3: 6 to 8 and INT4: Days 9 to 11).

**INT1**					
	**LR**	**SVM**	**NB**	**AdaBoost**	**GBT**
**Group A ***	0.69	0.73	0.63	0.72	0.77
**Group C ****	0.65	0.68	0.63	0.63	0.73
**Group D ****	0.73	0.76	0.76	0.72	0.77
**INT1-INT2**					
	**LR**	**SVM**	**NB**	**AdaBoost**	**GBT**
**Group A ***	0.72	0.74	0.66	0.73	0.79
**Group C ****	0.65	0.68	0.64	0.65	0.72
**Group D ****	0.74	0.76	0.77	0.71	0.77
**INT1-INT3**					
	**LR**	**SVM**	**NB**	**AdaBoost**	**GBT**
**Group A ***	0.73	0.74	0.66	0.71	0.77
**Group C ****	0.67	0.71	0.67	0.68	0.78
**Group D ****	0.74	0.78	0.76	0.73	0.79
**INT1-INT4**					
	**LR**	**SVM**	**NB**	**AdaBoost**	**GBT**
**Group A ***	0.73	0.73	0.71	0.76	0.77
**Group C ****	0.69	0.71	0.68	0.69	0.77
**Group D ****	0.75	0.79	0.79	0.70	0.81

* A stratified 10-fold cross validation procedure was used. ** Random downsampling with replacement was applied to match the control group with the target group due to the increased class imbalance (Section 2.1).
